# Beyond the Pale: Dark Traits and Close Relations Influence Attitudes toward COVID-19 and the Rejection of Quarantine Rules

**DOI:** 10.3390/ijerph18094838

**Published:** 2021-04-30

**Authors:** Pablo Espinosa, Miguel Clemente

**Affiliations:** Department of Psychology, Universidade da Coruña, 15071 A Coruña, Spain; miguel.clemente@udc.es

**Keywords:** dark traits, COVID-19, close relations, quarantine, moral disengagement

## Abstract

Dark personality traits are predictors of detrimental behavior (e.g., selfishness or violating norms). This research examined the influence dark personality traits on attitudes toward the COVID-19 pandemic and quarantine rules. We determined whether specific dark traits could predict non-compliance, beyond the global measure of dark personality traits. Additionally, previous research suggests that people are more likely to violate rules for the benefits of close relations, rather than for their own self-interests. We examined how this tendency interacts with dark traits. The 823 participants in the study completed measures of the dark triad, moral disengagement, and attitudes toward COVID-19 rules, and responded to vignettes about themselves or close relations escaping quarantine. Using a bifactor model approach, results showed that a general dark factor predicted non-compliance to COVID-19 rules, but that some moral disengagement mechanisms contributed to non-compliance beyond this factor. Vignette results showed that participants were more willing to break quarantine rules for a close relation than for themselves, except for those high in moral disengagement, who broke rules more—regardless of who was involved. These findings have important implications for intervention programs and policies, since individuals with dark traits tend to “selfishly” trespass norms, but anyone can “go beyond the pale, i.e., go outside the limits of acceptable behavior, for a loved one.

## 1. Introduction

It is estimated that approximately 20% of carriers of infectious pathogens cause 80% of new cases (for COVID-19 [1], as well as for any infectious disease [2]). Apart from biological causes and the environment, recent studies have investigated the role of behavioral patterns in facilitating the spread of COVID-19. Some studies researched the role of general personality variables, while others focused on personality traits related to harmful behavior toward others [3,4,5]. Individuals with a high level of dark traits disregard the negative effects of their behaviors toward others, show less empathy, and are less inclined to accept social norms. Hence, they are less likely to accept the restrictions imposed to curb the expansion of COVID-19, to the extent that they fail to perceive an imminent risk to themselves (or, if they do perceive it, they ignore it).

Previous research shows that individuals with high dark triad tendencies (i.e., Machiavellianism, narcissism, and psychopathy) are less compliant with isolation measures/less and COVID-19 restrictions [5,6,7]. In turn, Modersitzki et al. [4] state that individuals with high measures of psychopathy traits value anti-COVID-19 health rules less, perceive them to be more restrictive, ineffective, unsatisfactory, difficult to comply with, and are consistently less willing to accept them. These individuals also comply less with hygiene and distancing rules. Similarly, a high level of narcissism is related to disregarding leisure and social gathering restrictions, although no association was found with Machiavellianism. However, Blagov [3] found that non-compliance of COVID-19 social distancing and hygiene rules are related to psychopathy traits, and to a lesser extent Machiavellianism, but not to narcissism. Finally, Triberti et al. [8] found that individuals with high Machiavellianism, psychopathy, and to a lesser extent, narcissism, are less likely to comply with recommended COVID-19 behaviors (hygiene and reducing social interaction) and ignore rules in this respect. In short, current evidence shows that dark personality traits are significant predictors of whether individuals adhere to COVID-19 rules. However, evidence concerning the importance of each personality trait is still ambiguous, except perhaps in the case of psychopathy.

### 1.1. Dark Personality Traits

Overall, dark traits refer to subclinical personality traits that relate to ethically-, morally-, or socially questionable behaviors. High levels of dark traits are associated with selfishness or unfair decisions [9]. In addition, dark traits are associated with other personality variables, such as impulsivity, sensation seeking, short-term tactics, and risky behaviors [10].

Paulhus and Williams [11] coined the term “dark triad” to include three distinctive personality traits related to harmful behavior toward others: Machiavellianism, narcissism, and psychopathy. Machiavellianism entails a manipulative, cold, and calculating personality. Narcissism is a sub-clinical version of the personality disorder that maintains facets related to feelings of superiority, dominance, exaggerated importance, megalomania, needing admiration from others, a sense of entitlement, and lack of empathy. Psychopathy corresponds to a sub-clinical version as well, is associated with high impulsivity and sensation seeking, callousness, and a low level of empathy and anxiety. All of these traits share certain common characteristics, with different intensities. They are associated with behavioral tendencies that are harmful to others, personal promotion, emotional coldness, and aggression. However, they are distinct constructs that maintain correlations between 0.25 and 0.50. According to Jones and Paulhus [12], while psychopaths act impulsively, abandon their friends and families, and disregard their reputations, Machiavellians make plans, build alliances, and try to maintain good reputations. Narcissism is also characterized by being manipulative, but its haughtiness hides a sense of insecurity. It also differs from Machiavellianism in that it is non-deliberative and involves a certain level of self-deception. Narcissists tend to be optimistic, overconfident, and believe their exaggerations even when these are exposed. The motivation for narcissists is to increase their egos, while Machiavellians pursue instrumental motivations. In turn, the goals of Machiavellians are usually long-term, while a psychopath’s goals are often immediate. However, the three traits are characterized by callousness and manipulation of others. Subsequently, Paulhus [13] introduced the dark tetrad, adding sadism to the dark triad, which entails harming others for personal gain (pleasure).

Moshagen et al. [9] argue that dark personality traits have considerable overlap among them, and raise the notion of a common core to all of them (a general tendency to ethically-, morally-, or socially questionable behavior), named the Dark Factor of personality. According to his approach, dark personality traits are specific manifestations of a “general tendency to maximize one’s individual utility—disregarding, accepting, or malevolently provoking disutility for others—accompanied by beliefs that serve as justifications” (p. 657). When evaluating an action, individuals high in the “Dark Factor” do not give any value, or give a negative value, to the consequences for other people, but give a positive value to the results obtained for themselves. In turn, they maintain beliefs that serve as justifications for maximizing individual benefits at the expense of others. They may consider themselves superior, see others as inferior, or think that it is normal to behave as they do. Within this notion, any dark trait represents a particular facet of that common core, and although there are differences between them, they all reflect the same underlying general trend. Apart from the traits belonging to the dark tetrad, a wide variety of traits make up the Dark Factor of personality. Each trait, beyond the shared common core, have factors that make it unique. Additional dark traits included in the Dark Factor are selfishness (pursuing advantages or pleasure for oneself at the expense of others); sense of entitlement (the belief that one deserves more than others); self-interest (pursuing socially valued benefits); malevolence (a tendency to harm others even if it harms oneself); and moral disengagement (a cognitive orientation related to harmful behavior). In any case, the Dark Factor is understood as a fluid construct, independent of any dark trait and, thus, any given dark trait would be one indicator for this underlying core.

Among dark traits, moral disengagement deserves special attention. According to Bandura [14], as people develop moral identities, they adopt standards on what is right and wrong that guide or restrain their behaviors. Through a process of self-regulation, we contemplate our behaviors and evaluate it in relation to our moral principles, circumstances, and the consequences of our actions. We do things that increase our sense of integrity and refrain from violating our moral principles to avoid self-deprecation. Bandura et al. [15] argue that people self-regulate their behaviors when they assess that it is violating, or will violate, their internal rules or standards. Breaching personal standards of behaviors creates an aversive state for the individual that triggers control processes to avoid the violation. However, these control processes must be activated to operate, but sometimes self-regulation is suspended because a process of disengagement from internal controls is activated. Individuals can avoid this aversive state when committing harmful behavior by reinterpreting their behaviors, disguising their personal agencies, minimizing, or ignoring the negative consequences, or blaming the recipients of their behavior. Within the first set of mechanisms, focused on reinterpreting harmful behavior, in positive terms, are moral justifications, euphemistic labeling, or advantageous comparisons. Moral justification entails explaining harmful behavior as morally positive, and personally or socially acceptable. Euphemistic labeling involves using convoluted or “sweetened” language to describe reprehensible behavior and present it as benign. Advantageous comparison involves contrasting reproachable behavior with a more serious transgression, so that it seems irrelevant or even positive in comparison. Other mechanisms entail distorting the behavioral agency through displacement or dissemination of responsibility. Displacement of responsibility involves attributing responsibility of the harmful act to the situation or others, so that, since agency for the behavior is diminished, one can avoid self-censorship more easily. Diffusion of responsibility involves accepting only an irrelevant role in a harmful action, or attributing it to a collective decision. Any negative action committed by one’s group is likely to be attributed mainly to others through this mechanism. The third group of mechanisms implies ignoring or distorting the consequences of the action, focusing on its positive effects (personal gain, for example), while minimizing negative effects for the victims. Finally, disengagement mechanisms can target the victim or recipient of harmful behavior. One of these mechanisms is dehumanization. When we perceive someone as an individual like us, it is difficult not feeling empathy for the individual’s discomfort—or harm the individual without feeling bad. Dehumanization is achieved by stigmatizing victims and attributing negative traits that make them worthy of negative behavior, and it prevents us from perceiving them as people with the same feelings, desires, and concerns we have. Another mechanism related to victims is attribution of blame. Through this mechanism, victims are given responsibility for the harm they suffer, and this harm is construed as a retaliation caused by some behavior performed by the victim.

Moral disengagement mechanisms do not automatically make the individual insensitive and cruel. As the self-censorship caused by minor transgressions diminishes, the individual is able to increasingly tolerate harmful behaviors, until they stop causing discomfort [14]. People use these mechanisms as neutralization techniques; that is, rationalizations that define acts contrary to their own moral principles as appropriate in a particular situation. This allows people feeling committed to their moral principles while carrying out behaviors that violate them. The motivation to act against moral principles would arise from the discrepancy between the personal cost of violating the principles and the cost of losing the potential benefit that the action could have. At least in situations related to small acts of social deviation, it is common for people to distort the moral implications of the behaviors they wish to perform and act against their principles [16]. Moral disengagement is different from other dark traits in that it focuses on avoiding internal sanctions associated with violating moral principles. For other dark traits, beliefs related to the justifications of harmful behaviors are more general (cynicism, maximizing profits at the expense of others, sense of superiority, etc.), and individuals with these beliefs do not deny that the behavior is harmful, but justify it in terms of the personal outcomes obtained. People who avoid internal sanctions using disengagement strategies are acting in their own interests, but need to rationalize their immoral behaviors, and cannot ignore his moral principles when they conflict with the desired outcomes. The discrepancy between the behaviors and the principles they breach cause an aversive state, so individuals are motivated to distort their cognitions in order to achieve a rationalization that reconciles their principles with the behaviors that violate them. Therefore, moral disengagement can be the dark trait that better explains harmful behavior when individuals are concerned with maintaining their perceptions (i.e., that they are wholly, morally people).

### 1.2. Rule Breaking and Close Others

In addition to transgressions committed for selfish reasons, and for personal gain, it is also interesting to examine both harmful behaviors committed by close relations and transgressions committed to benefit them.

Van Prooijen [17] states that moral judgments depend on social closeness to the perpetrator of a harmful act. Compared to out-group offenders, judgments of blame for in-group offenders are usually more benevolent when guilt is not certain. This “benefit of doubt” that applies when the guilt of the perpetrator is uncertain, may also apply to the outcomes of a transgression when they are not evident. Social closeness is an important factor influencing the assessment of moral transgressions. When a close relation commits a transgression (compared to a stranger), it is perceived as less serious, more forgivable, and less selfishness; more repentance is attributed to the perpetrator, and there is greater willingness to help the perpetrator [18].

In addition, Cadsby et al. [19] state that when someone cheats, it is not always for a selfish reason. Sometimes an individual commits a transgression to benefit a close relation, and although this is often detrimental to unknown people, victims and consequences are not acknowledged. They found that people cheat both for their own sakes and to benefit their own groups. However, the moral burden associated with selfish transgressions that dissuades individuals to cheat for their own benefit is absent when it comes to favoring socially close people. A transgression to benefit someone else is more likely to happen than a selfish transgression, because it is less likely to be perceived (or self-perceived) as selfish or socially rejected behavior. It may even be positively evaluated and foster a perception of power in the transgressor. In this sense, Van Kleef et al. [20] argue that powerful individuals are more likely to break the rules to take a chance to achieve their goals. Socially unacceptable behaviors can increase an individual’s perception of power if they benefit their own groups rather than themselves. Breaching rules for the benefit of others is perceived as a “prosocial” violation of rules, and the offender is likely to be perceived as having a higher level of power.

### 1.3. Objectives and Hypotheses

The objectives of this research were two-fold. First, we examined how individuals with dark traits responded to the COVID-19 pandemic and complied with quarantine rules. We also examined whether individuals were more willing to violate quarantine norms when it benefitted close relations (rather than themselves), and how this interacted with dark traits.

Thus, one major objective was to examine the effects of the shared common core of dark traits, defined as the Dark Factor, and compliance with COVID-19 rules. Moreover, we examined whether some specific dark traits explained attitudes toward COVID-19 beyond the Dark Factor. As dark personality traits often involve a component of impulsivity and lack of planning, the role of this variable, as a predictor of risky and antinormative behaviors, was also examined. Additionally, since males score higher in antinormative behavior and dark traits [15,21], and in “bad behaviors” related to COVID-19 [8], sex differences in dark traits and COVID-19 rules compliance were examined.

Another objective was to evaluate differences between breaking health rules, committed by individuals themselves versus close relations, and rule breaking for personal gain or for the benefit of close relations. Previous research shows that when a close relation benefited, the likelihood of committing a transgression increased, in comparison to transgressions for personal gain [19], because the moral stigma of committing a selfish act was not perceived. This can lead to a higher likelihood of breaking health standards when they affect loved ones. We will also examined whether there were differences when non-compliance created a direct risk (for oneself or a close relation) as opposed to a more general and diffuse risk.

Specifically, the following hypotheses are drawn:

**Hypothesis 1** **(H1).** *The Dark Factor of personality will positively predict attitudes and behaviors related to COVID-19.*

**Hypothesis 2** **(H2).** *Specific dark traits, related to moral disengagement strategies, will positively predict COVID-19-related attitudes and behaviors, independently of the effects of the Dark Factor.*

**Hypothesis 3** **(H3).** *Participants will be less likely to break health related rules when the risk directly affects them or close relations.*

**Hypothesis 4** **(H4).** *Participants will be more likely to break health related rules to benefit close relations, compared to breaking rules for their own benefit.*

**Hypothesis 5** **(H5).** *There will be an interaction between transgressions committed for personal gain vs. close relations and dark traits. Participants with low dark traits will show a higher likelihood of favoring close relations compared to favoring themselves, whereas this discrepancy will be reduced for participants high in dark traits (since acting selfishly is less of a concern for them).*

## 2. Materials and Methods

### 2.1. Sample

The study sample initially involved 894 participants from northwestern Spain, but it was necessary to eliminate 71 participants on suspicion of careless responding; thus, the final sample was 823. The mean age of the participants was 31.30 years (*SD* = 15.03; range: 18–70) and two thirds were female (65.7%). Data were collected between October and November 2020. In terms of COVID-19 infection: 2.3% of participants had been previously infected or were infected at the time of participating in the study, and 0.7% preferred not to answer this question. These data were consistent with the levels of contagion in the population in Spain during data collection dates. In addition, 26.5% of participants knew people close to them who were infected (0.6% preferred not to answer this question).

### 2.2. Instruments

Participants were first asked to respond to a vignette about breaching a quarantine to escape an epidemic. To avoid the influence of previous attitudes, beliefs, and controversies related to the current pandemic that could affect responses, the disease was portrayed as an unknown and extremely serious illness, so that the need for quarantine could not be questioned, and biased beliefs would be less likely to obscure the influence of vignette manipulations of the participants’ responses.

The situation was manipulated so that the protagonist of the story would be the participant, or someone close to him. In addition, either the risk of non-compliance with the quarantine was personal or general. In the personal risk condition, non-compliance affected the participant directly and the close relation took refuge in the participant’s house, and vice versa. In the general condition this was not mentioned, so the risk was diffuse and affected the population in general. This way, combining these variations—four “between subjects” conditions were designed: protagonist: self vs. close relation by risk: personal vs. general).

Participants were previously asked to choose someone they felt very close to from the following list: mother, father, sibling, child, partner, or best friend. Whomever they chose was later the character in the vignettes for participants in the “close relation” conditions.

The following vignette corresponded to the “self” condition. Italics include the phrase that appeared only in the personal risk condition:

[The quarantined island]

“Imagine the following situation is real:

You are living in an island and there is an epidemic outbreak of a contagious disease that is lethal in 80% of cases. Currently no cases have been reported outside the island and the authorities decide to declare a quarantine and forbid anyone in or out of the island. You consider escaping to avoid getting sick with this dangerous disease. The island is close to the mainland and you could leave on a boat with no risk of being seen, although you should do it right away, because later you may not have the chance. [*Then, on the mainland, you would take shelter in your (close relation choice) house.*] You are currently in good health, although you do not know if you may be carrying the disease with you.”

For the participants who responded to the vignette involving a close relation, the story was equivalent, but the main character was the previously chosen close relation. In the personal risk condition, the selected character took refuge in the participant’s house.

Participants were then asked how likely they would be to leave the island (or tell their close relation to do so), whether it would be understandable to leave the island, and they were asked to make a final decision in qualitative terms (leaving/leaving but taking great care/not leaving). These questions constituted the dependent variables for the vignette.

Participants completed measures of moral disengagement, the dark triad of personality, impulsivity, and attitudes and behaviors related to COVID-19 rules.

As a measure of moral disengagement, we used the Moore et al. [22] Propensity to Morally Disengage (PMD) scale, which is 24 items long and an adaptation for the general population of the Moral Disengagement Scale (MDS) by Bandura et al. [15]. It measures the eight mechanisms of moral disengagement, described by Bandura, in addition to offering an overall index on a scale of 7 points (1: totally disagree–7: totally agree). The reliability of the PMD in our sample was high (α = 0.89). This scale was translated to Spanish and back translated by bilingual researchers; no differences in meaning were detected.

Jones and Paulhus’ SD3 (Short Dark Triad) scale [11] was used to measure the dark triad, involving 27 items measuring Machiavellianism, narcissism, and psychopathy, using a 5-point scale (1: totally disagree–5: totally agree). The SD3 questionnaire showed acceptable overall reliability in our sample (α = 0.82), while the scales for each of the dark triad variables showed a somewhat lower reliability (Machiavellianism, α = 0.73; narcissism, α = 0.63; psychopathy, α = 0.69). This scale as adapted to Spanish by Pineda et al. [23].

Psychopathy includes a component of impulsivity, so we considered including an impulsivity questionnaire not related to psychopathy, as this is a relevant variable for explaining risky behaviors. The BSSS-4 (Brief Sensation Seeking Scale-4) and SS2 (Sensation Seeking-2) scales described by Stephenson et al. [24] were chosen due to their consistency and brevity (four and two items, respectively). Participants responded using a 1 (never) to 5 (very often) scale. Both scales were combined on an impulsivity scale for the purposes of this study; its reliability was 0.84. The procedure to translate impulsivity scales was the same used for the PMD scale.

In addition, participants completed a scale created for this study on COVID-19 attitudes and behaviors. It included items related to the perception of the pandemic (e.g., “Current measures for the prevention of COVID-19 are excessive”); behavioral intentions (e.g., “If I started having symptoms of COVID-19, I would wait a few days to see if it is some other illness, to avoid having to quarantine”) and behaviors related to COVID-19 (e.g., “When I wear a face mask I place it under my nose or chin to be able to breathe better”). We used a 7-point response scale (1: totally disagree/never–7: totally agree/very often). The reliability of this scale and its factor analysis are described in the results.

### 2.3. Procedure

Data collection was carried out using online surveys. University students were provided the option to collaborate in data collection in exchange for course credits. Collaborators were asked to contact voluntary participants they were acquainted with and provide them with a link that gave access to the study. This allowed us to gather a wider variety of participants, as opposed to merely contacting participants of a particular internet social network. The link contained a brief description of the study, and by clicking it, they were assigned to the different experimental conditions using a randomization programming script. After reading a broader description of the tasks to complete, and providing their informed consent, they were presented with the vignettes and the scales corresponding to the covariates of the study.

In studies that use surveys, there is a risk that participant responses will be inconsistent and contain errors due to careless responding, tiredness, or lack of motivation to provide accurate answers, especially when there is a considerable number of questions. Online surveys, compared to written surveys, allow mechanisms to improve the quality of answers (such as avoiding missing responses). However, the ease of answering in this format also allows participants to quickly mark answers without properly looking at questions. Following Maniaci and Rogge [25], we included two key items in the survey that required a specific answer (“I read the instructions carefully. To show that you have read these instructions, mark number 1”; and “This is a control question, please mark number 5 and continue.”) All participants who failed to give the required answers to both questions were removed from the final sample.

This research complied with the Helsinki Protocol [26] criteria and APA ethical standards [27].

### 2.4. Data Analysis

First, we calculated the reliability of the measures used in the study and split the sample in two parts to carry an exploratory and confirmatory factor analysis of the questionnaire on attitudes toward COVID-19. Gender differences and correlations between variables were also examined. Using a bifactor model design, we checked whether dark personality traits had a differential effect on attitudes toward COVID-19 rules beyond their contributions, to a general effect determined by the Dark Factor of personality. Finally, an ANCOVA was conducted to determine the effect of the vignette IVs and covariates on participants’ responses.

## 3. Results

### 3.1. Descriptive Analysis and Gender Differences

Participants’ scores in dark traits were, on average, low (moral disengagement: *M =* 2.32, *SD =* 0.81; Machiavellianism: *M =* 2.73, *SD =* 0.70; narcissism: *M =* 2.50, *SD =* 0.62; psychopathy: *M =* 1.83, *SD =* 0.61). The average impulsivity score was close to the midpoint of the scale range (*M =* 2.77, *SD =* 0.86). In regards to COVID-19 attitudes and behaviors, the scores showed that participants, on average, complied with norms (*M =* 2.19, *SD =* 0.85).

As for vignette responses, there was, on average, a higher chance of asking a close relation to leave the island (*M =* 4.57, *SD =* 2.07) compared to leaving oneself (*M =* 3.22, *SD =* 1.91). To a lesser extent, the same was true for the question of whether it would be understandable to leave the island (close relation: *M =* 4.30, *SD =* 1.79; self: *M =* 4.87, *SD =* 1.76. In this case, low scores indicated that leaving was perceived as more understandable).

As Table 1 shows, notable differences were found between groups in qualitative decisions related to hypothetical scenarios (χ^2^(6) = 79.656, *p <* 0.001). Leaving the island, regardless of whether one took precautionary measures, was a transgression. By adding up both options, the percentage of norm violations in favor of a close relation was around 70% compared to 40% when the character in the vignette was the participant.

In regards to gender differences, Table 2 shows that males scored significantly higher than females in all dark traits and impulsivity. In addition, males scored higher in negative attitudes and behaviors related to COVID-19. Females only scored significantly higher on the likelihood of leaving, or asking a close relation to leave the quarantine in the vignette, although there were no differences with males in perceiving this transgression as understandable by others.

### 3.2. COVID-19 Questionnaire Factor Analysis

The sample was randomly split into two halves for the exploratory (EFA) and confirmatory factor analysis (CFA) carried out on COVID-19 attitudes and behaviors. Initial EFA results using the Varimax rotation showed that all items except one (“I comply with COVID-19 prevention rules even if I think they are excessive or uncomfortable”) loaded above 0.40 on a single factor. Subsequently, following Pituch and Stevens [28], this item was dropped, and the resulting EFA explained 34.79% of the variance with a single factor, with factor loads ranging from 0.427 to 0.715. The KMO factor showed an appropriate value (0.88), as well as Bartlett’s test of sphericity (χ^2^(105) = 1857.470, *p* < 0.001). Table 3 showed the factor loads for each item within this general factor.

On the other hand, the CFA also showed that the single factor solution had a good fit (χ2(51) = 64.20, *p* = 0.101; RMSEA = 0.025 [CI: 0.001–0.042]; SRMR = 0.0335; CFI = 0.99. The reliability of the overall scale was very acceptable (α = 0.84).

### 3.3. Bifactor Model

**Figure 1:**
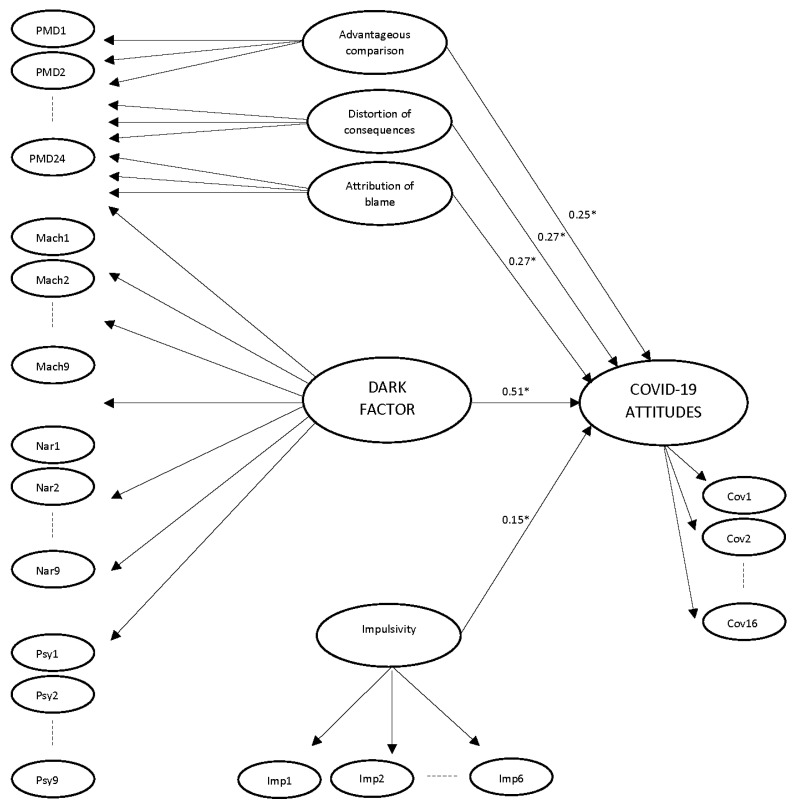
Bifactor model for predicting attitudes toward COVID-19. * p < 0.05. Standardized total effects.

A SEM bifactor model to predict COVID-19 attitudes and behaviors was tested. In a bifactor model, each indicator loads on a general first order factor, to capture the common variance shared by all items, and also on a first order specific factor, which captures the variance explained exclusively by the variable this factor represents [29,30]. The specific factors explain the remaining variance that is not explained by the common general factor. Although we did not include, in this study, all of the different traits that comprise the Dark Factor, it is a robust construct, independent of specific traits, so it does not affect its predictive validity [13]. To perform this analysis, we designed a bifactor SEM model using AMOS 24, where all dark trait indicators loaded on a general factor used as a proxy for the Dark Factor of personality, and on specific first order factors with predictive power. Because the sample size [31] and the model size [32] influence χ2, and both are high in our study, we used other goodness-of-fit statistics (SRMR and RMSEA). All dark trait indicators loaded onto a first-order Dark Factor, and in the specific first-order factors that retained predictive power for attitudes toward COVID-19 rules, independent of the general Dark Factor, as Figure 1 shows:

Although χ^2^ is significant, due to the sample and model size, other indices show that the model has a good fit (χ2(2470) = 8890.99, *p <* 0.001; RMSEA = 0.056 [CI: 0.055–0.057]; SRMR = 0.082). The Dark Factor, which represents the common core to all dark traits, was a good predictor of COVID-19 attitudes and behaviors, as Hypothesis 1 stated. In addition, other variables related to the Dark Factor were specific predictors of attitudes toward COVID-19 beyond the Dark Factor. These predictors, as expected in Hypothesis 2, were moral disengagement strategies: advantageous comparison, distortion of consequences, and attribution of blame; which are likely to relate to the specific excuses that an individual may use to justify non-compliance with the rules on COVID-19 prevention. Finally, impulsivity was also a predictor of attitudes and behaviors versus COVID-19. Psychopathy had a component of impulsivity, but this model suggests that the effect of psychopathy on attitudes and behaviors toward COVID-19 is explained through the Dark Factor common core. It was, therefore, congruent that impulsivity, independent of psychopathy, was a significant predictor of attitudes toward COVID-19.

### 3.4. Correlations

As shown in Table 4, responses to the quarantine vignette correlated with COVID-19 attitudes and behaviors. In addition, they correlate with Machiavellianism, narcissism, and moral disengagement. COVID-19 attitudes and behaviors showed significant positive correlations with impulsivity and dark personality traits, especially moral disengagement and psychopathy. Correlations between dark traits were also high and they correlated with impulsivity, especially psychopathy.

### 3.5. Likelihood of Breaching the Quarantine

An ANCOVA was conducted to determine the effect of experimental conditions and covariates on the likelihood of breaching the quarantine in the study vignette. As opposed to what we expected in Hypothesis 3, initial analyses showed that there were no significant main effects for the risk IV (general risk vs. personal risk), so only the protagonist IV (self vs. close relation) was considered in the final model. We did not find a main effect for age either, so it was also dropped from the analysis. As for the chosen close relation, which subsequently was the character in the vignette in the close relation condition, 40.2% of participants chose their mother, 15.9% their sibling, 14.2% their child, 13.6% their partner, 10% their father, and 6.2% their best friend.

Sex, dark traits, and impulsivity were used as predictors. The model obtained was significant (adjusted *R*^2^ = 0.165; *F*(8, 822) = 21,327, *p <* 0.001, η^2^ = 0.173) and Table 5 summarizes the effects found for the IV and covariables in the model.

**Figure 2:**
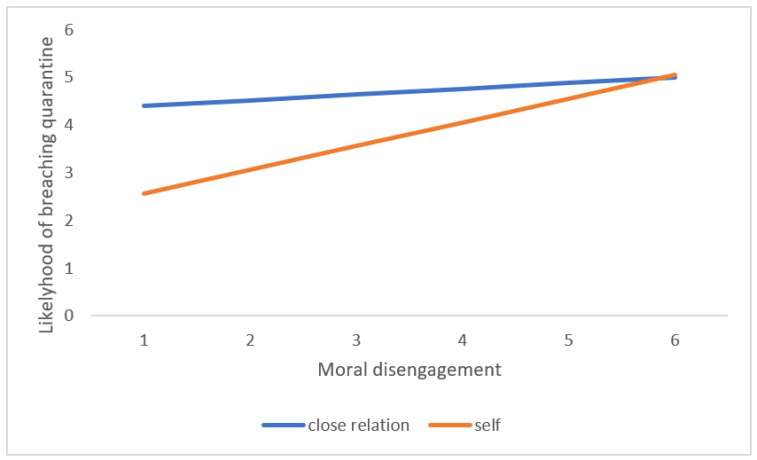
Interaction between protagonist IV and moral disengagement.

Consistent with Hypothesis 4, main effects were found for the protagonist IV (Self *M* = 3.22, *SD* = 1.91 vs. Close relation *M* = 4.57, *SD* = 2.07). Most covariates also showed a significant main effect. The main effect for sex showed that females were more likely to break the quarantine than males (male *M* = 3.53, *SD* = 2.14 vs. female *M* = 4. 09, *SD* = 2.06). Among dark traits, the biggest main effect was for psychopathy, followed by Machiavellianism and moral disengagement, while narcissism only showed a marginally significant main effect. Impulsivity also showed a main effect. In addition, as Hypothesis 5 predicted, an interaction effect was found between the protagonist IV and moral disengagement. This interaction showed that, while the probability of breaching the quarantine when the character in the vignette was the participant depended on his/her level of moral disengagement; asking a close relation to break the quarantine remained high, irrespective of the level of moral disengagement, as Figure 2 shows.

## 4. Discussion

Results confirm that the common core of dark traits, conceptualized as the Dark Factor, predicts attitudes and antinormative behaviors related to COVID-19, which is consistent with previous research [5,6,7]. Although some research highlights the importance of some dark traits over others [3,4], they do not consider the common effects captured by the Dark Factor. This common core integrates the general tendency of dark traits to show a lack of empathy and disregarding negative outcomes for others while maximizing positive results for oneself. Still, some specific moral disengagement strategies predict attitudes toward COVID-19 independently of this common core. These strategies are consistent with plausible justifications for antinormative behavior in relation to COVID 19:

- “I do not break COVID-19 rules as much as others” (advantageous comparison).
- “Other people break COVID-19 rules too, so I am not the only one” (advantageous comparison).
- “The risk of getting sick is very small” (distortion of consequences).
- “My behavior only affects me” (distortion of consequences).
- “In most cases COVID-19 has no serious consequences” (distortion of consequences).
- “If someone gets COVID-19 it is because they have done something to get infected” (attribution of blame).
- “It’s up to everyone to be protected from COVID-19 if they want, and I don’t have to worry about others” (attribution of blame).

The existence of a general Dark Factor, predicting attitudes toward COVID-19, confirms Hypothesis 1 of the study, while the specific strategies that predict these attitudes, irrespective of the common core of dark traits, are consistent with Hypothesis 2. In addition, all dark traits in the study have a significant effect on quarantine breaking in the vignette, which provides additional support for the role of dark personality traits on health rules violations.

As for Hypothesis 3, results were not as expected. The personal risk condition showed no significant differences from the general risk condition. Table 1 shows nearly identical scores for leaving the island irrespective of the risk condition. When the personal risk was for the participant, non-compliance with the quarantine may even be misperceived as an altruistic sacrifice rather than as a transgression, as the participant accepts sheltering a potential carrier of a deadly disease. Results show a small decrease in quarantine breaching when it involved putting a close relation at risk, but the ANCOVA results were non-significant.

Regarding the qualitative decision about the vignette, the option of leaving, but taking precautions, was chosen by many more participants than just leaving, probably because it allowed participants to keep thinking they would be committing a less flagrant transgression or because it provided a mechanism to justify the transgression (e.g., “I skipped quarantine, but it is ok because I was careful”).

Nevertheless, a very relevant main effect for the protagonist IV was found for the decision to breach quarantine. Participants were much more willing to break the rules for the benefit of a loved one than for themselves. As Cadsby et al. [19] state, breaching health rules is a transgression nevertheless, but when it benefits a loved one, individuals have a moral alibi that helps them perceive they are not acting selfishly—or that they are even acting altruistically, giving the proper advice, or making a sacrifice for the other person. It may even increase the sense of power in the individual through the perception of being a person with initiative and determination, as van Kleef et al. [20] suggests regarding rule violations that benefit others. In addition, results show an interaction effect between the vignette character and moral disengagement that supports Hypothesis 5. All participants showed a high level of non-compliance when the protagonist was a close relation, irrespective of their score in moral disengagement. However, while participants with low moral disengagement scores on average indicated a low probability of breaking the quarantine when they were the characters in the vignette, individuals with a high level of moral disengagement stated that they would probably break it irrespective of who was the protagonist. This indicates that most people low in moral disengagement tend to commit more transgressions for the benefit of a close relation rather than for personal gain. This likely happens because it is easier to justify internally and externally a transgression that may be construed as “altruistic.” However, individuals who show a high level of moral disengagement have an easier time justifying a selfish act and choose to pursue their own benefit as often as when they can justify a “prosocial” transgression. They are more used to cognitive strategies that mitigate the aversive feelings of committing a transgression and break rules, regardless of whether it benefits them or a close relation without the need for further motivations.

Impulsivity was included as a relevant variable in the explanation of risky behaviors and this research shows it is a significant predictor of anti-normative behavior, independent of dark personality traits, but with less explanatory power. By comparison, dark traits are much stronger predictors for this type of behavior, so our results highlight the importance of dark traits to predict this type of transgressions. Results for sex showed the expected relationships, but it should be noted that although men scored higher in dark traits, impulsivity, and antinormative attitudes toward COVID-19, women scored higher in the likelihood of breaching the quarantine in the vignette.

## 5. Limitations and Future Directions

Among the limitations in this study is the type of sample used, which was incidental, and it greatly limits its generalizability. Additionally, two-thirds of the sample were female, which also limits generalizability. As an attempt to increase the validity of the sample, instructions were given to the collaborators to contact mainly people from their daily environment, rather than people they only knew online. Another limitation is that data collection was based on self-reports. In any case, at the dates when data collection took place, it would not have been possible to proceed otherwise because of the health risk regulations. However, the sample size and the criteria used to ensure the quality of the data described in the procedure contributed to the validity of the results. Given the magnitude of cognitive and personality variables that may be relevant to explain the behaviors that can facilitate the transmission of infectious diseases, all research on this matter is inevitably partial. However, it may be interesting to consider other general personality variables, and variables related to coping and stress. Other issues that can be explored are environmental variables that influence perceived risk, perceived severity, degree of negative consequences certainty, or the individual’s degree of involvement in antinormative behavior.

## 6. Conclusions

These results may be useful in the design of intervention programs to achieve greater adherence to community rules related to health risks. The general influence of dark personality traits is confirmed, but in addition, this study reveals some specific traits that are associated with non-compliance beyond the Dark Factor. This may help design specific interventions to counter cognitive strategies that foster transgression. In this sense, moral disengagement mechanisms can be disabled using arguments highlighting hypocrisy to neutralize the excuses or justifications used to commit transgressions, through a cognitive restructuring and attitude change process.

More importantly, interventions should contemplate that people are willing to break rules to a greater extent if they believe it is for the good of someone they feel close to. Our results show that participants violate rules even more to benefit close relations than when it involves personal gain---probably under the impression that transgressions that benefit close relations are “prosocial” or “altruistic” or that they show loyalty or commitment. We must consider that the sacrifices and rules individuals embrace for themselves may be rejected when they affect a close relation. Taking health risks may even be perceived as the right thing to do when it involves close relations. Thus, attitude change programs should contemplate that changes should focus not only on the behavior of individuals, but also on the behavior toward their close social network and the cognitive biases that may affect their decision-making regarding close relations.

Finally, although non-compliance behaviors are performed by individuals, authorities should consider these social behaviors when designing their policies. In this sense, authorities should not ignore that a certain proportion of people are prone to reject health-related rules and develop adequate policies to decrease health risks. Knowing the cognitive and personality variables associated to an increased health risk is essential in this respect.

## Figures and Tables

**Table 1:** Qualitative response to the scenario in each condition.

VI Protagonist	Close Relation		Participant	
VI Risk	General Risk	Personal Risk	General Risk	Personal Risk
Staying in the island	31.5%	29..1%	56.2%	61.2%
Leaving the island, but being careful	57.3%	56.8%	41.4%	35.3%
Leaving the island	11.2%	14.1%	2.4%	3.5%

**Table 2:** Sex differences.

	*t*	Males		Females	
		*M*	*SD*	*M*	*SD*
Probability of leaving	3.64 *	3.53	2.14	4.09	2.06
Leaving is understandable	0.13	4.60	1.86	4.58	1.76
COVID-19	4.81 *	2.39	0.91	2.09	0.80
Impulsivity	5.01 *	2.98	0.86	2.66	0.85
Moral disengagement	5.01 *	2.51	0.87	2.22	0.76
Machiavellianism	5.47 *	2.91	0.71	2.64	0.67
Narcissism	4.17 *	2.63	0.65	2.44	0.59
Psychopathy	8.64 *	2.08	0.67	1.70	0.54

* *p <* 0.001. *d. f. =* 821.

**Table 3:** Factor weights of attitudes and behaviors related to COVID-19 (scale 1–7).

	*M*	*SD*	*Factor Loading*
1. Current measures for the prevention of COVID-19 are excessive.	2.31	1.66	0.694
2. Rules for COVID-19 are so strict that I am not surprised that people do not follow them.	2.06	1.62	0.714
3. There is no point in worrying much about COVID-19.	1.58	1.15	0.715
4. If I were quarantined but I needed to leave the house, nothing would happen if I went out and came back quickly.	2.00	1.56	0.567
5. If I were infected with COVID-19, I would avoid reporting close contacts if doing so could cause any inconvenience to them.	1.63	1.36	0.549
6. COVID-19 is not as contagious or serious to take so many measures to prevent it.	1.60	1.38	0.703
7. I do not believe that I am personally at risk of contracting COVID-19.	2.00	1.55	0.594
8. If I got COVID-19, it would most likely be asymptomatic.	3.20	1.89	0.457
9. If someone infected me with COVID-19 and then I infected someone else, the blame would be on the person who infected me in the first place.	1.90	1.51	0.491
10. When unfortunately, someone dies from COVID-19 it is probably because they would have died soon anyway because of their underlying conditions.	1.98	1.46	0.660
11. If I started having symptoms of COVID-19, I would wait a few days to see if it is some other illness, to avoid having to quarantine.	2.14	1.63	0.615
12. In the street and public places, I wait and give other people priority to keep social distancing (reversed).	5.56	1.60	0.427
13. When I wear a face mask, I place it under my nose or chin so I can breathe better.	1.58	1.15	0.553
14. I have attended meetings or events where I did not follow the existing norms at that time for the prevention of COVID-19.	2.35	1.57	0.484
15. When I am with friends or family and they remove their mask, even if it is against the rules, I take it off too.	3.82	2.10	0.511

**Table 4:** Correlations between variables in the study.

	Probability of Leaving	Leaving Understandable	COVID-19	Impulsivity	Disengagement	Machiavellianism	Narcissism
Leaving understandable	−0.28 **						
COVID-19	0.17 **	−0.07 *					
Impulsivity	0.06	0.05	0.34 **				
Disengagement	0.10 **	−0.11 **	0.55 **	0.30 **			
Machiavellianism	0.12 **	−0.10 **	0.36 **	0.21 **	0.61 **		
Narcissism	0.10 **	0.03	0.30 **	0.34 **	0.30 **	0.39 **	
Psychopathy	−0.02	−0.04	0.47 **	0.52 **	0.54 **	0.47 **	0.38 **

* *p* < 0.05; ** *p* < 0.01.

**Table 5:** ANCOVA for the likelihood of breaching the quarantine.

	*F*	η^2^
Protagonist IV	28.410 **	0.034
Sex	20.023 **	0.024
Psychopathy	15.469 **	0.019
Machiavellianism	9.657 *	0.012
Narcissism	3.611 ^+^	0.004
Psychopathy	15.469 **	0.019
Moral disengagement	6.010 *	0.007
Impulsivity	5.942 *	0.007
Moral disengagement X Protagonist IV	4.136 *	0.005

* *p* < 0.05; ** *p* < 0.001; ^+^ = 0.06. *d. f.* = 1, 822.

## Data Availability

Data for this study are available online at https://doi.org/10.6084/m9.figshare.14132984.v1 (accessed on 29 April 2021).
